# Supplementary material for: Melatonin as a Repairing Agent in Cadmium- and Free Fatty Acid-Induced Lipotoxicity
Source: Biomolecules. 2023 Dec 7;13(12):1758. doi: 10.3390/biom13121758 (PMC10741790; doi:10.3390/biom13121758)

Original immunoblots Figure 3

A) Phospho-ERK1/2

Chemiluminescence

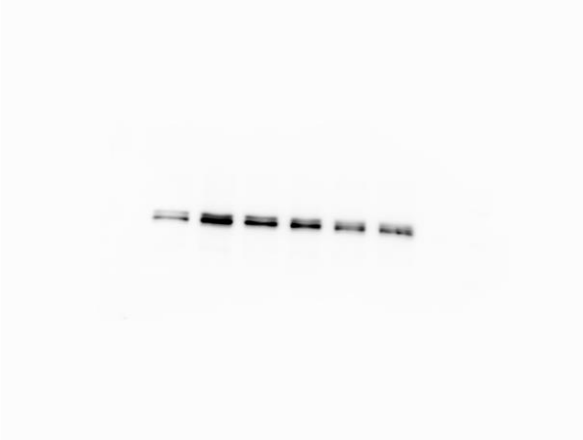

Colorimetric (merge)

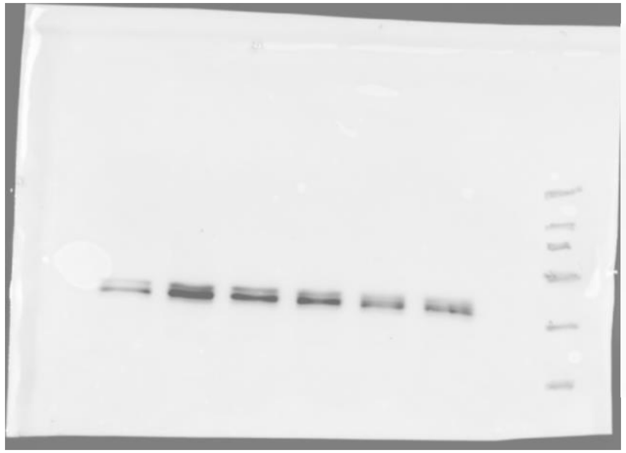

B) Phospho-SAPK/JNK

Chemiluminescence

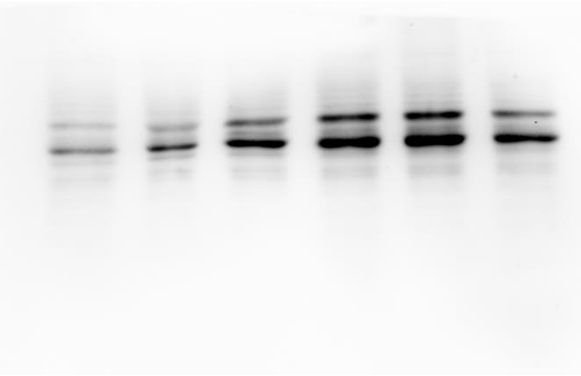

Colorimetric (merge)

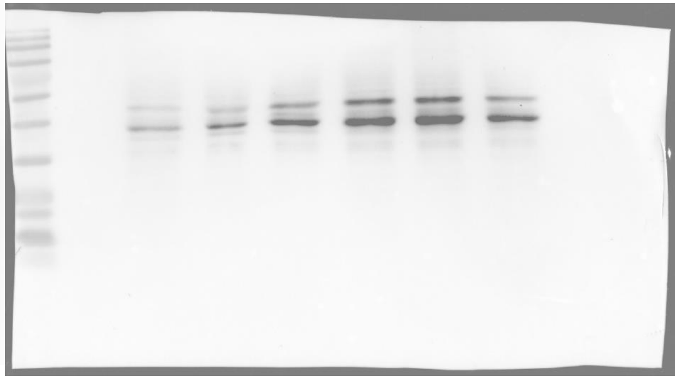

A) ERK1/2

Chemiluminescence

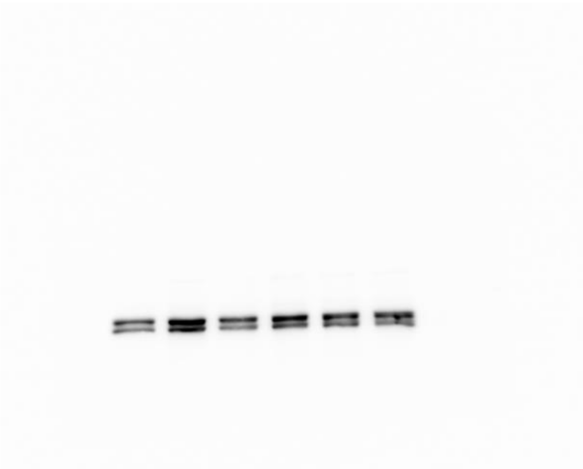

Colorimetric (merge)

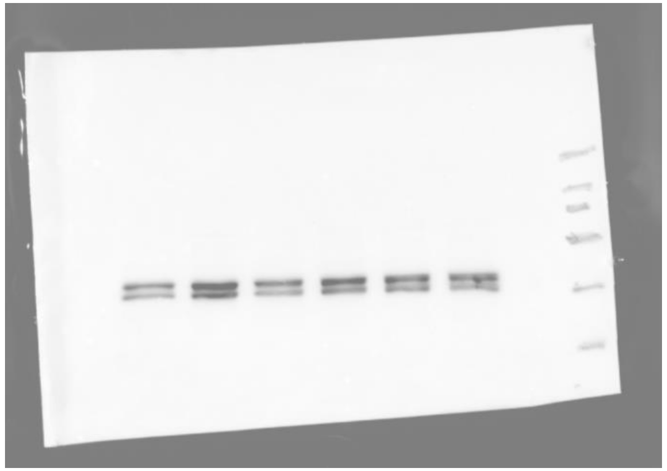

B) SAPK-JNK

Chemiluminescence

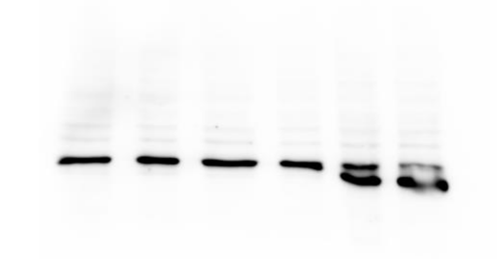

Colorimetric (merge)

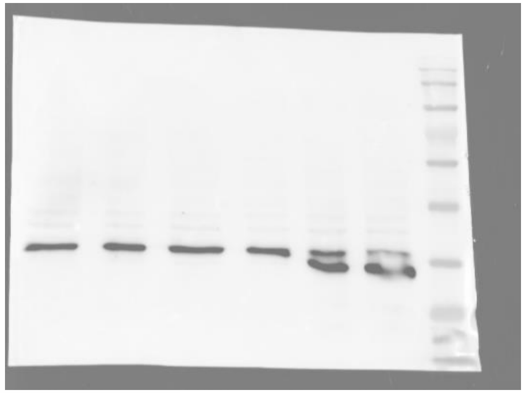

Original immunoblots Figure 3

C) Phospho-p38

Chemiluminescence

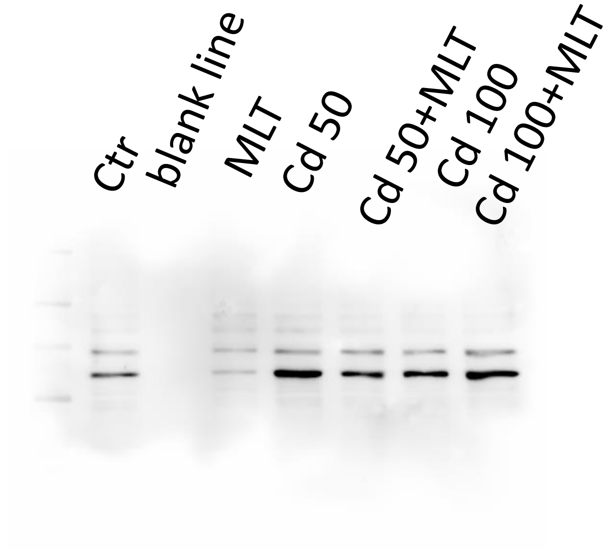

Colorimetric (merge)

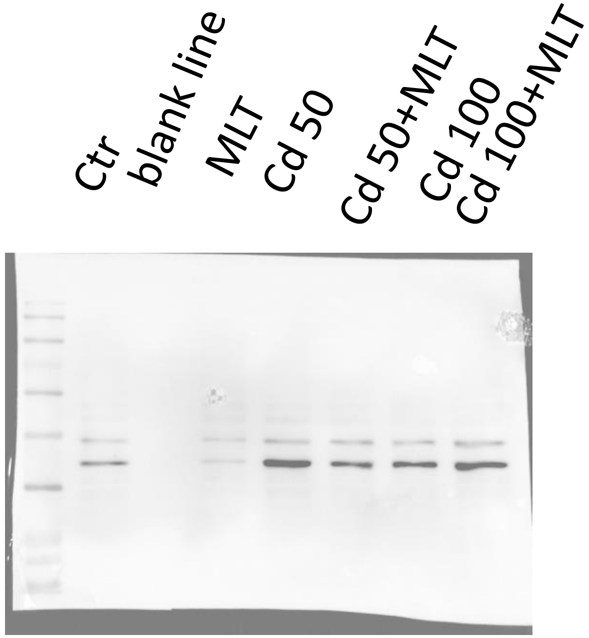

C) p38

Chemiluminescence

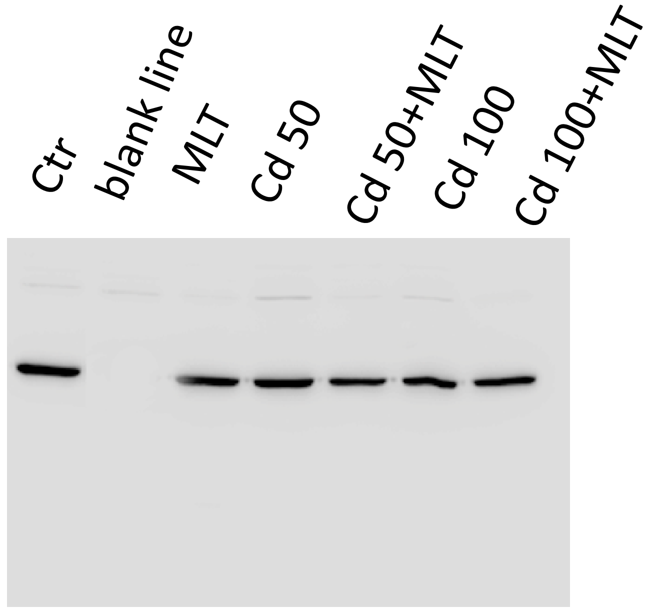

Original immunoblots Supplementary Figure S3

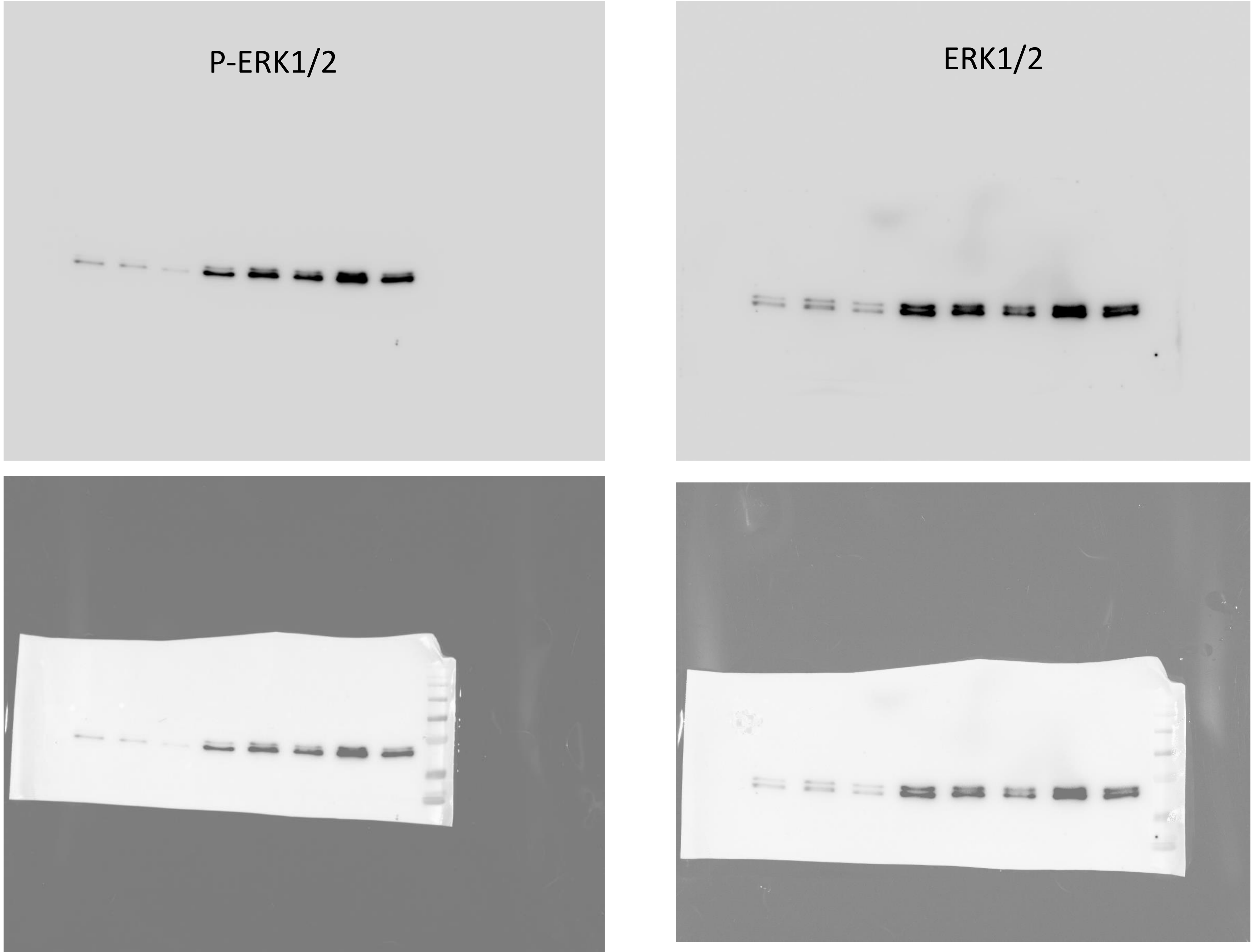

Original immunoblots Supplementary Figure S4

A) CAT

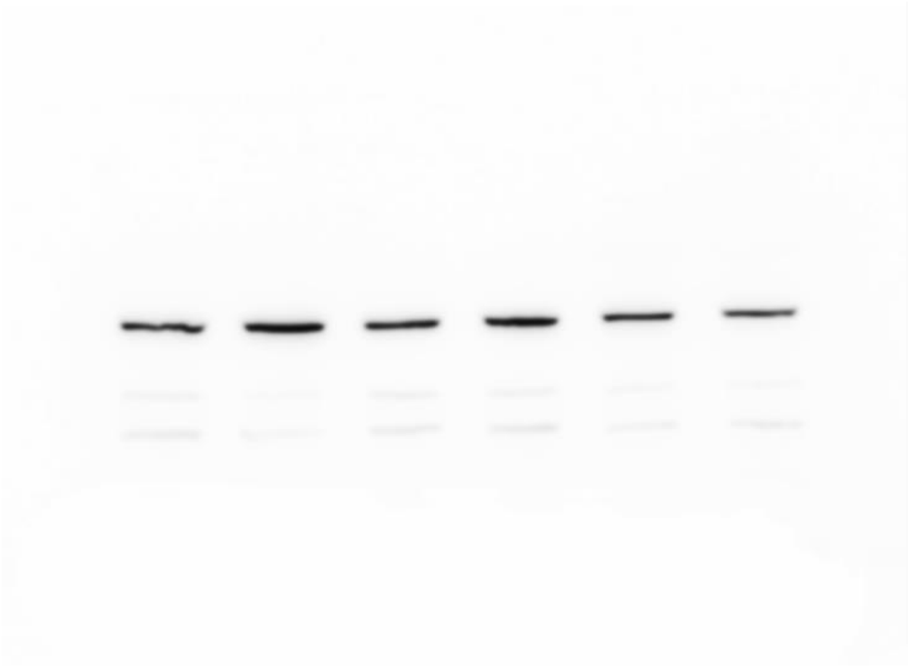

A) SOD2

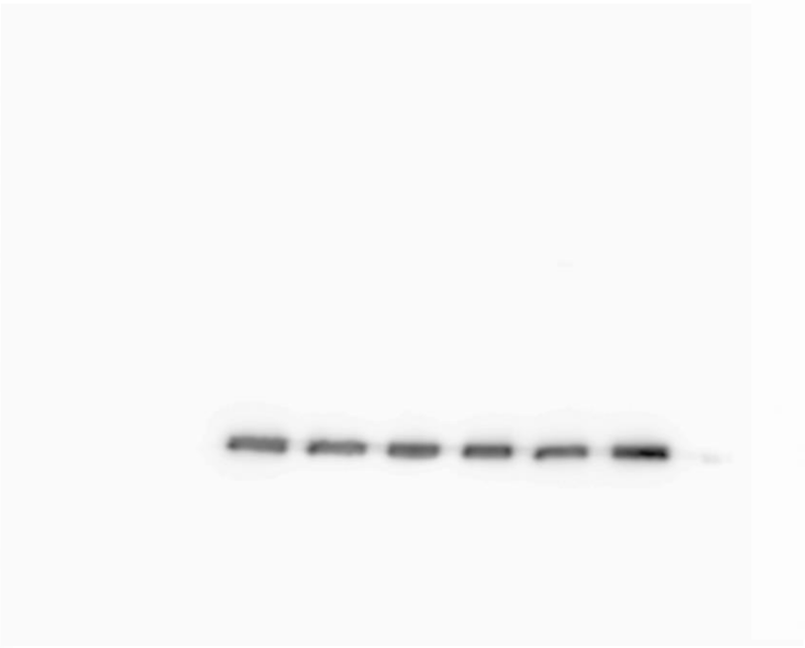

A) GAPDH

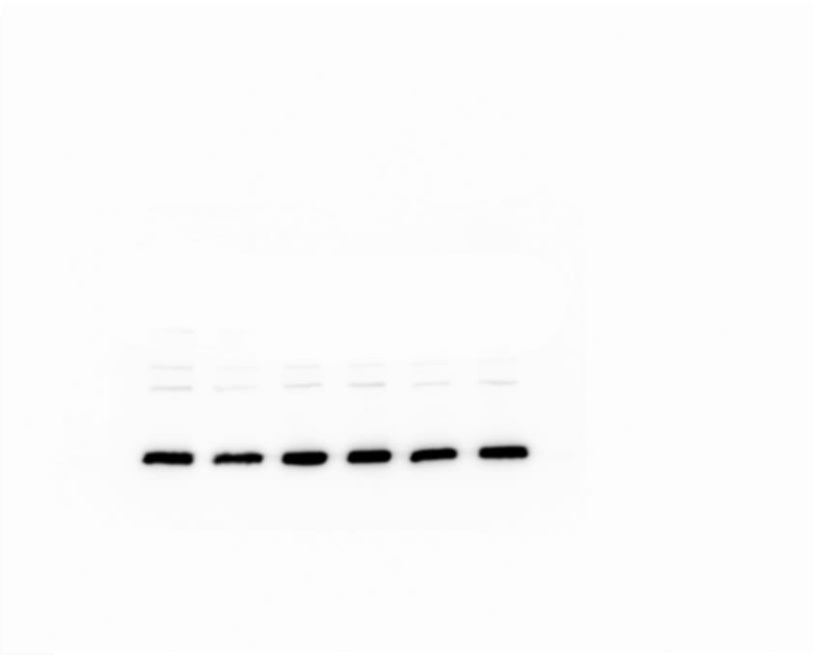

C) CAT

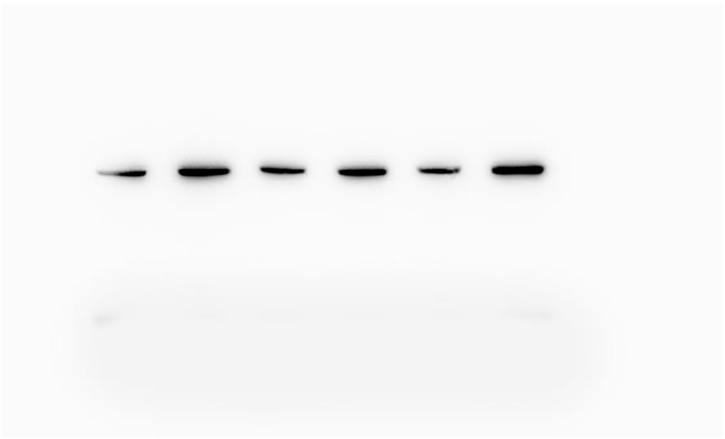

C) SOD2

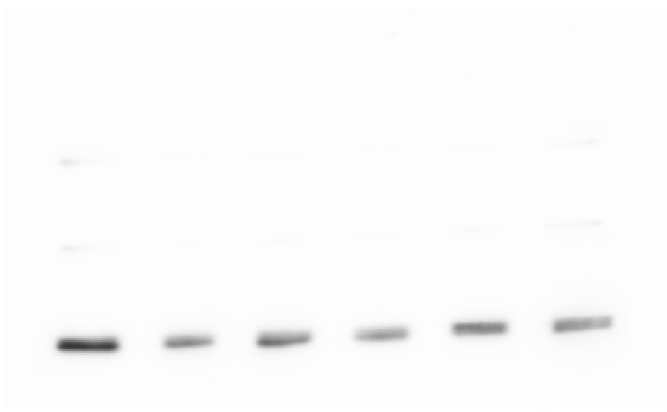

C) GAPDH

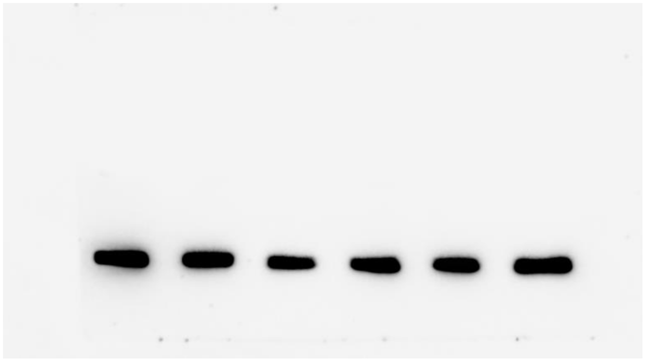

Original immunoblots Supplementary Figure S5

A) 5-LOX

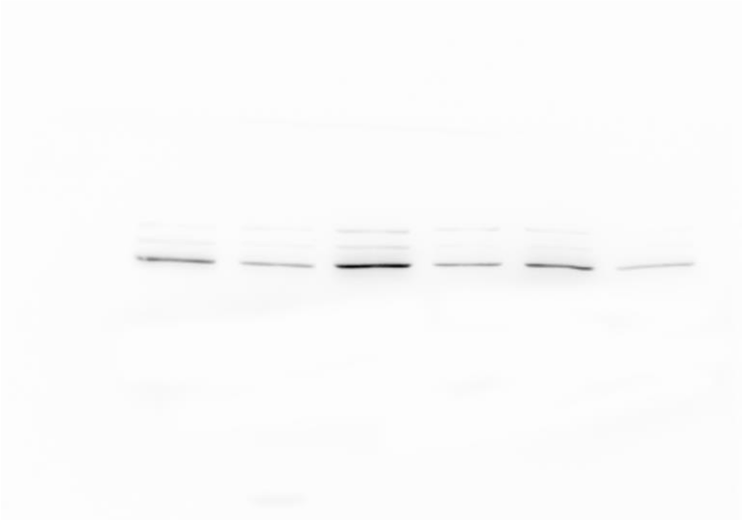

A) β-Tubulin

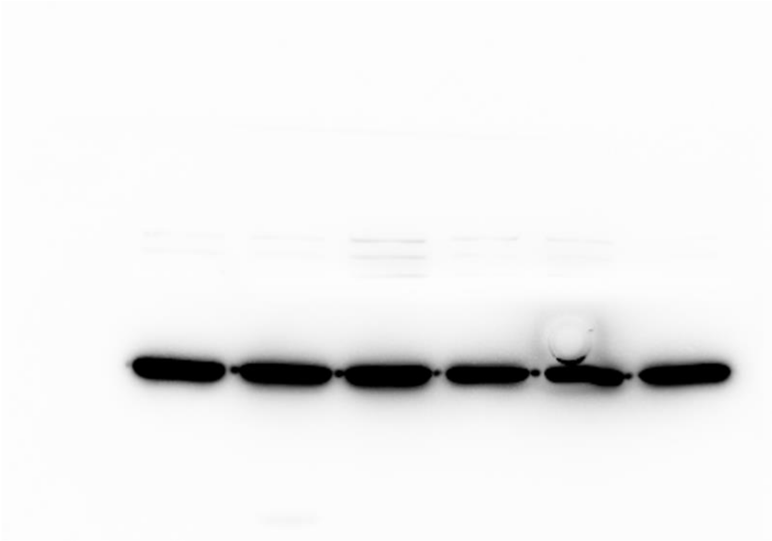

B) 5-LOX

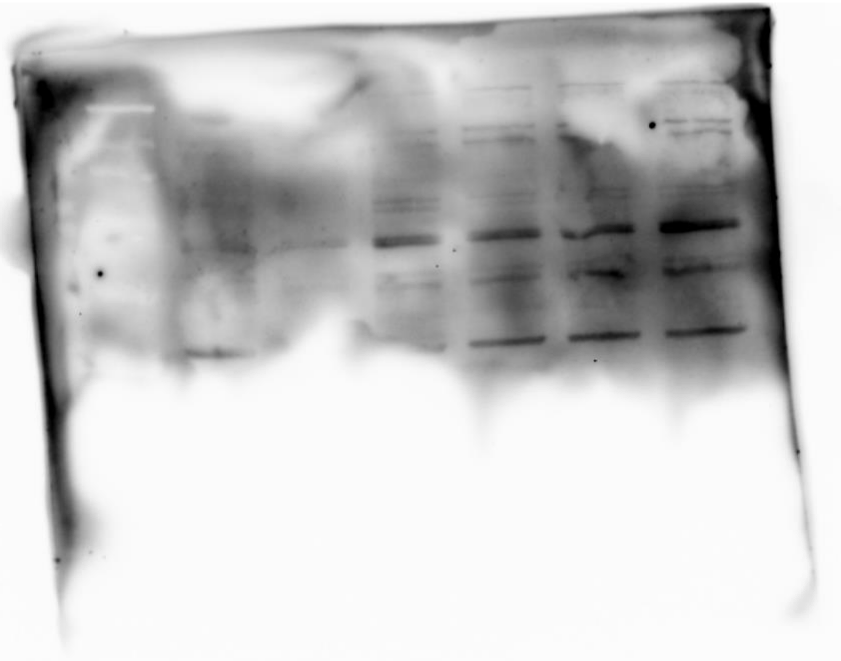

B) β-Tubulin

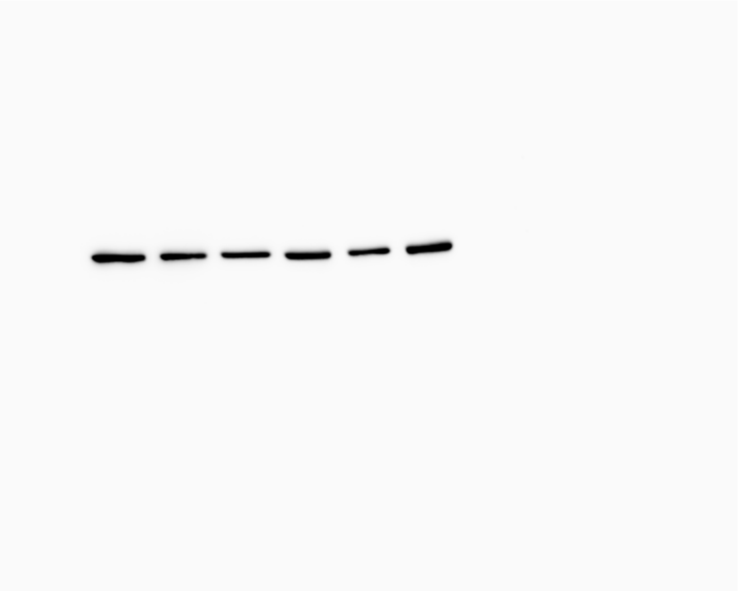

Supplement: Supplementary file 1 [file biomolecules-13-01758-s001.zip › biomolecules-2617721-original WB images.pdf]
