# Supplementary material for: Melatonin as a Repairing Agent in Cadmium- and Free Fatty Acid-Induced Lipotoxicity
Source: Biomolecules. 2023 Dec 7;13(12):1758. doi: 10.3390/biom13121758 (PMC10741790; doi:10.3390/biom13121758)
Supplement: Supplementary file 1 [file biomolecules-13-01758-s001.zip › biomolecules-2617721-supplementary.pdf]

Supplementary materials

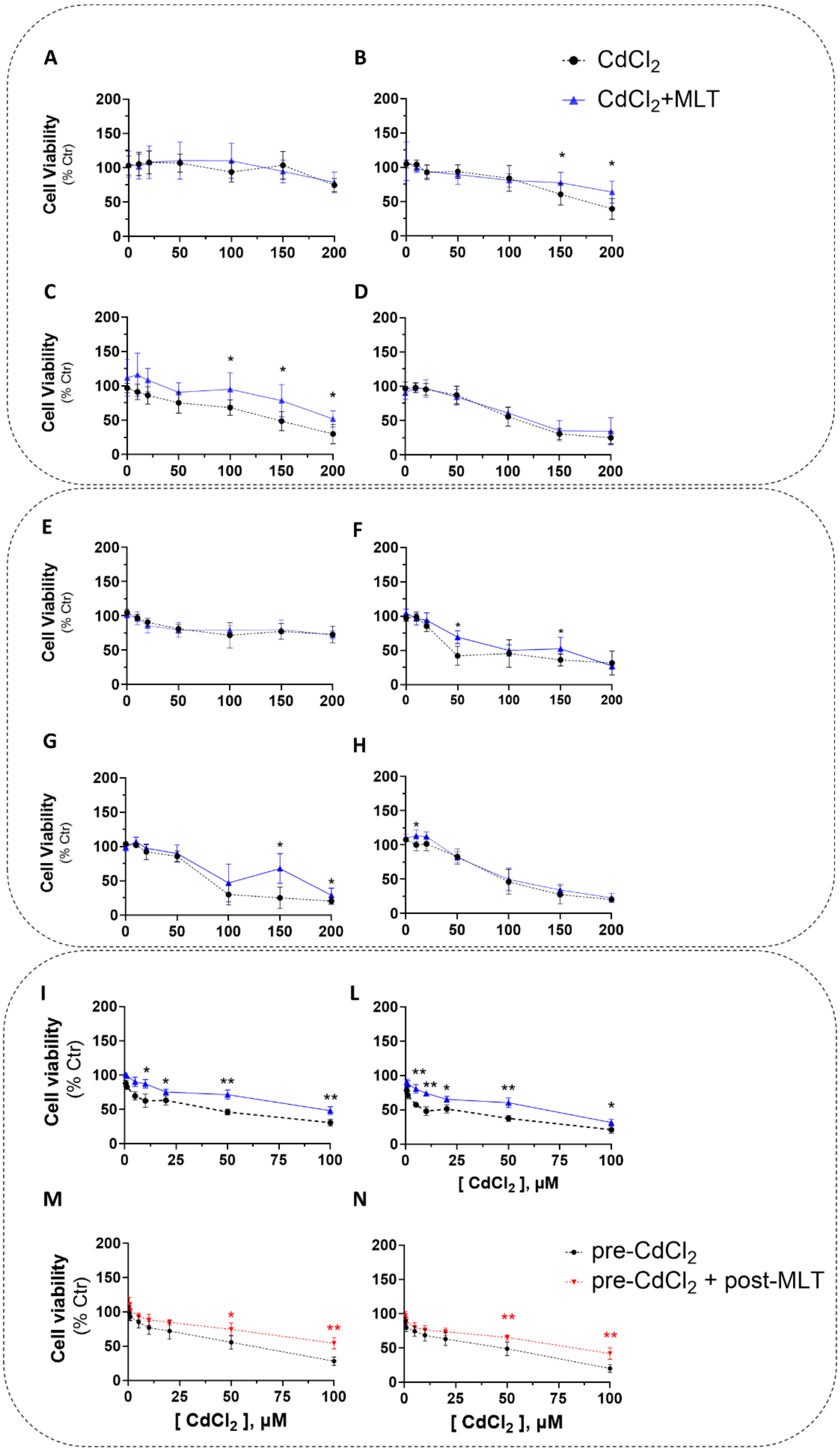

Supplementary Figure S1. Concentration- and time-dependent effect of $CdCl_2$ and cytoprotective effect of MLT on cell viability of HepaRG cells (A-D), CACO-2 cells (E-H) and primary murine hepatocytes (I-N). The cell tests were performed using the EP1 of Figure 1. (A and E) 3h, (B and F) 24h, (C, G and I) 48 h and (D, H and L) 72h. primary murine hepatocytes were pre-treated with CdCl2 24h and then treated with 50 nM MLT for 24 h (M) or 48h (N). One-way ANOVA test. *p<0,05; **p<0.01 (CdCl2 vs. CdCl2+MLT or pre-CdCl2 vs. pre-CdCl2+ post MLT).

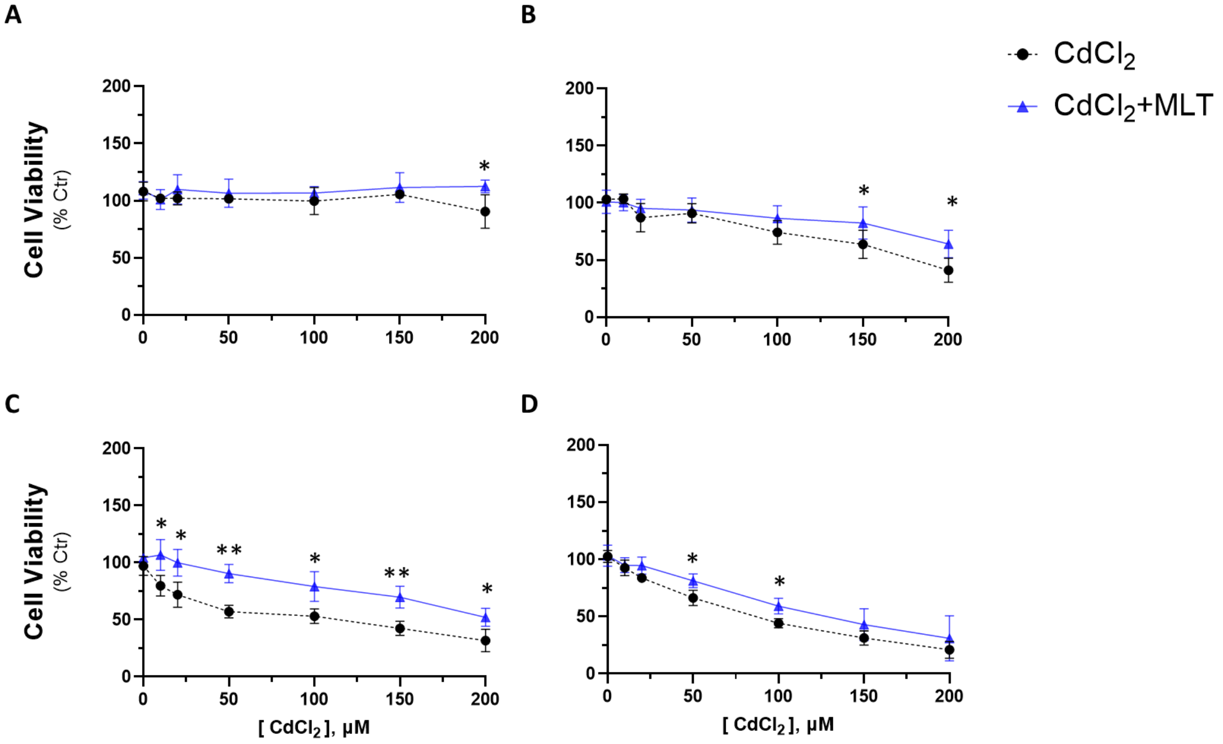

Supplementary Figure S2. Concentration- and time-dependent effect of CdCl2 on cell viability of HepaRG cells differentiated to hepatocyte-like cells, and cytoprotective effect of MLT. The cell tests were performed using the EP1 of Figure 1. (A) 3h, (B) 24h, (C) 48 h and (D) 72h. One-way ANOVA test. *p<0,05; **p<0.01 (CdCl2 vs. CdCl2+MLT).

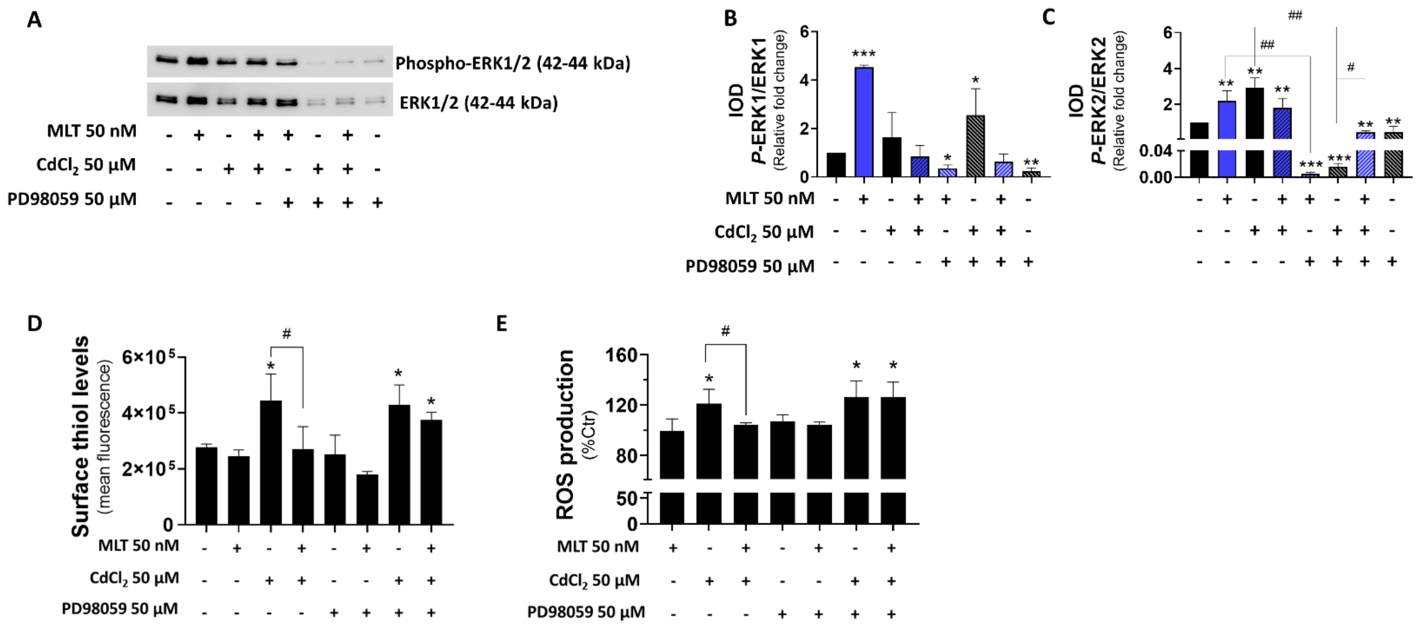

Supplementary Figure S3. Effects of MAPKs ERK1/2 activity MLT-related in HepaRG cells pre-treated with ERK inhibitor and treated with $CdCl_2$ and MLT. (A-C) MAPK-ERK1-2, (D) surface thiol levels and (E) ROS were evaluated in HepaRG cells after cells pre-treatment with ERK inhibitor (PD98059) 1h and then treatment with $CdCl_2$ (50 µM) and MLT (50 nM) for 3h. One-way ANOVA test: *p<0.05; **p<0.01; ***p<0.0001 (control vs. all treatments). #p<0.05; ##p<0.01 ($CdCl_2$ vs. all treatments).

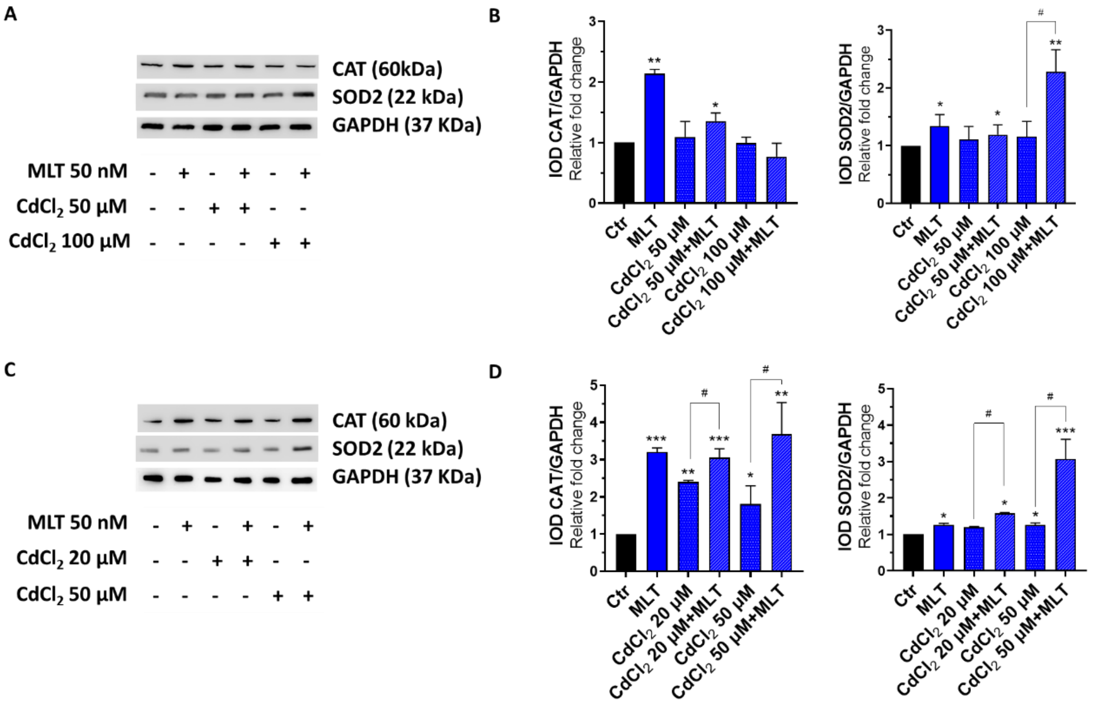

Supplementary Figure S4. Immunoblotting of antioxidant enzymes in HepaRG (A-B) and CACO-2 (C-D) cells treated for 24hrs with $CdCl_2$ and/or MLT. Relative optical density of the immunoblot bands as relative fold change of Ctr is represented in panel B and D. CAT, catalase; SOD, superoxide dismutase. *p<0.05; **p<0.001; ***p<0.0001 (Ctr vs. all treatments); #p<0.05 ($CdCl_2$ vs $CdCl_2$+MLT).

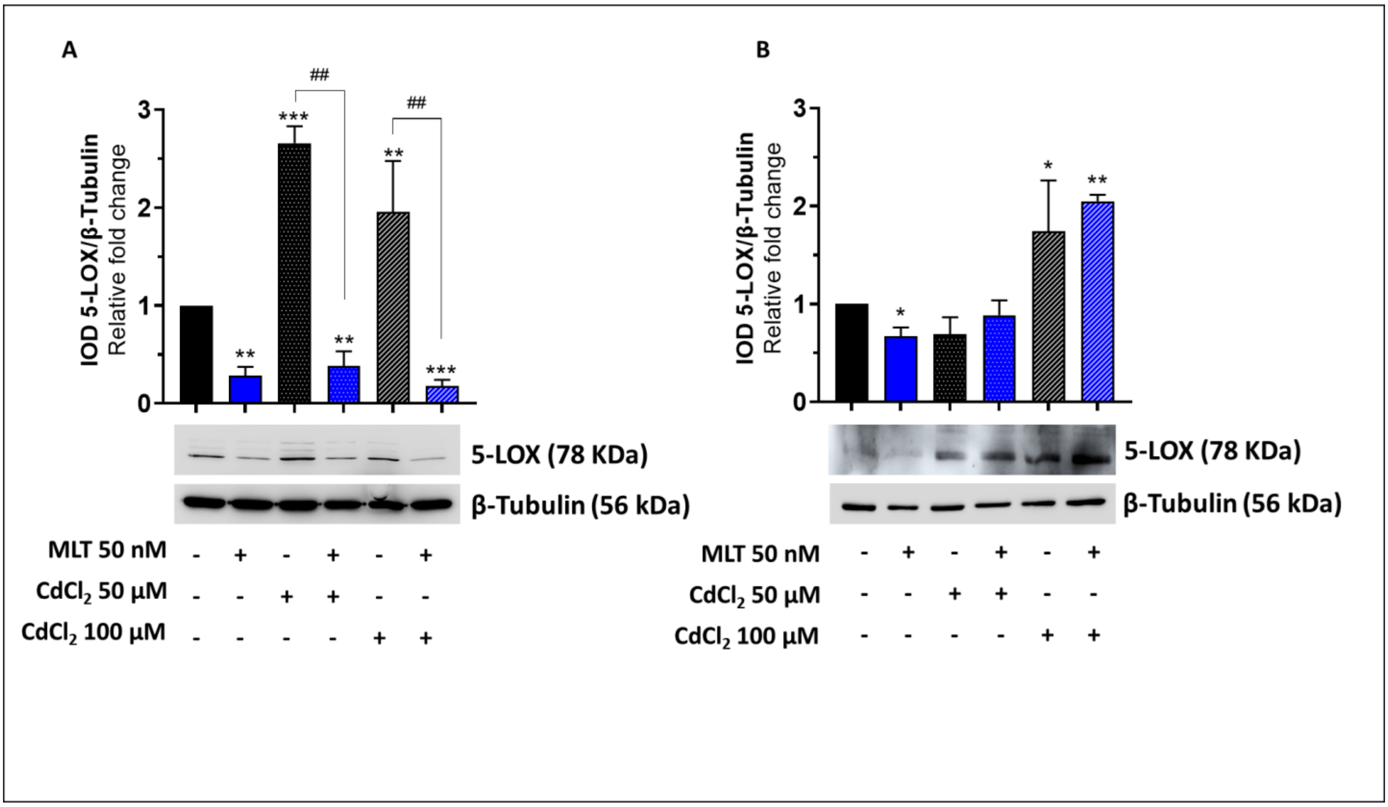

Supplementary Figure S5. Immunoblotting of 5-LOX in HepaRG (A) and CACO-2 (B) cells treated for 4 hrs with $CdCl_2$ and/or MLT. Relative optical density of the immunoblot bands as relative fold change of Ctr is represented. *p<0.05; **p<0.001; ***p<0.0001 (Ctr vs. all treatments); ##p<0.01 ($CdCl_2$ vs $CdCl_2$+MLT).

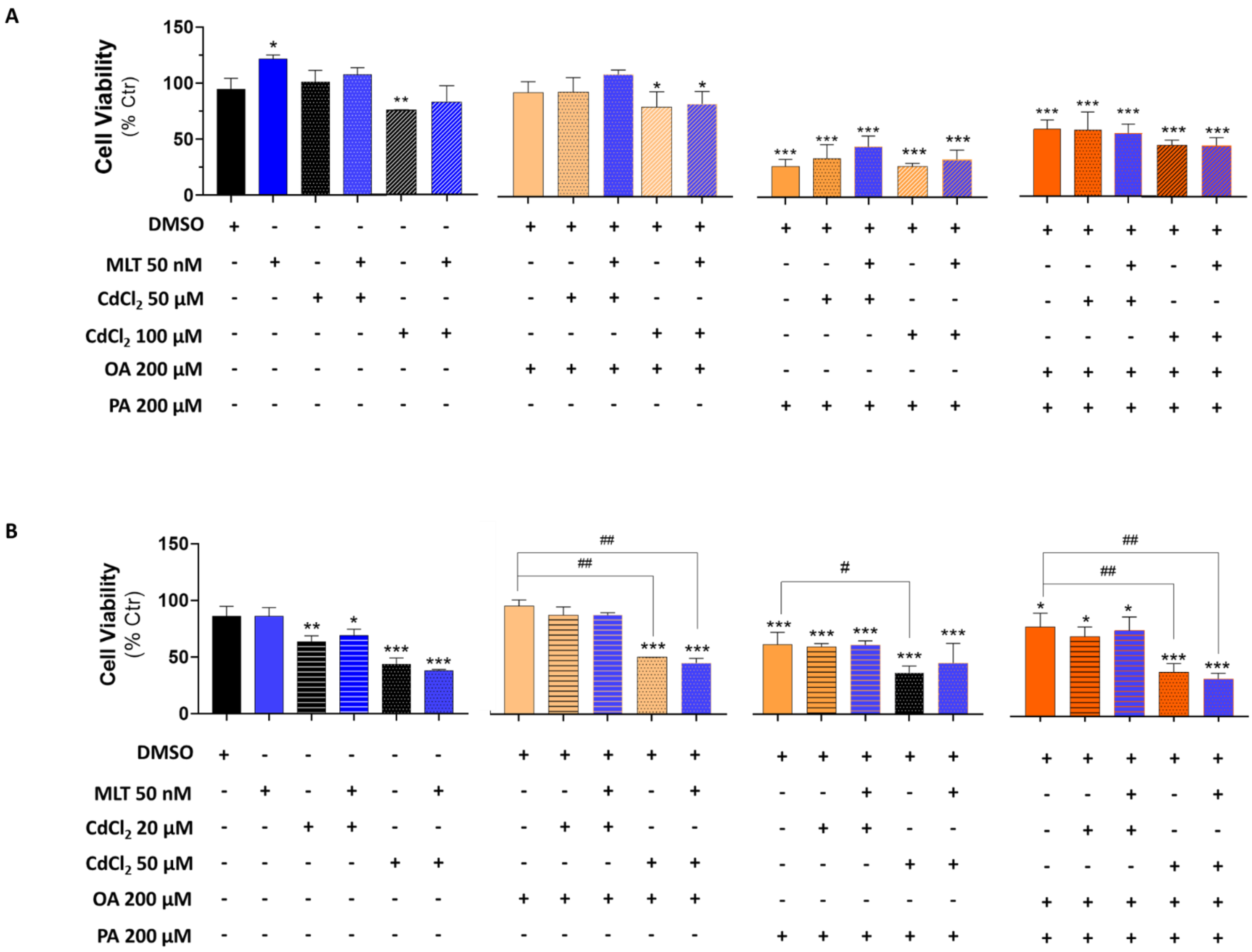

Supplementary Figure S6. Effect of $CdCl_2$, FFA and MLT on cell viability of the human liver cell line HepaRG and in CACO-2 intestinal cells. The effect of Cd and FFA on cell viability of HepaRG (A) and CACO-2 (B) cells was studied with EP2 (Figure 1), in which after 24-h pre-treatment with 50 or 100 µM $CdCl_2$ for HepaRG cells and 20 and 50 µM $CdCl_2$ for CACO-2 cells, these were treated for 48 h with FFAs (200 µM) and/or the cytoprotective agent MLT (50 nm). One-way ANOVA test: *p<0.05; **p<0.01; ***p<0.001 (control vs. all treatments). #p<0.05; ($CdCl_2$ vs. all treatments); #p<0.05; ##p<0.01 ($CdCl_2$ and/or FFAs vs. $CdCl_2$ and/or FFAs + MLT).
